# Global burden of childhood developmental intellectual disability caused by iodine deficiency from 1990 to 2021 and projection to 2040

**DOI:** 10.3389/fpubh.2025.1598949

**Published:** 2025-07-09

**Authors:** Zihao Liu, Youhui Lin, Li Liang, Xuanyi Li, Zhiyin Wang, Wei Cheng

**Affiliations:** ^1^School of Traditional Chinese Medicine, Beijing University of Chinese Medicine, Beijing, China; ^2^School of Management, Beijing University of Chinese Medicine, Beijing, China

**Keywords:** iodine deficiency, developmental intellectual disorder, Global Burden of Diseases, childhood, Bayesian age-period-cohort model

## Abstract

**Background:**

Iodine deficiency remains a leading preventable cause of childhood developmental intellectual disability (DID), imposing a substantial and enduring global public health burden; despite decades of global efforts to combat iodine deficiency, persistent health inequalities and uneven progress highlight critical gaps. This study aimed to comprehensively analyze the global burden, temporal trends, and inequalities in childhood DID attributable to iodine deficiency from 1990 to 2021 and projected to 2040. Our results will inform evidence-based public health policies, especially in the most affected areas.

**Methods:**

This observational study utilized secondary data from the Global Burden of Disease 2021 study, which covered 204 countries and territories. The burden of DID was stratified by age, sex, and region. Age-standardized prevalence rates (ASPR) and age-standardized years lived with disability (ASYR) were calculated. Trends were analyzed using joinpoint regression by estimated the annual percent change and average annual percent change (AAPC). Health inequalities were assessed using the slope index of inequality. Forecasts to 2040 were generated using the Bayesian age-period-cohort model.

**Results:**

Global ASPR declined from 43.06 to 8.96/100,000 (AAPC = −4.95) and ASYR from 7.71 to 1.67/100,000 (AAPC = −4.81) between 1990 and 2021. Despite this progress, low socio-demographic index (SDI) regions, particularly Central Sub-Saharan Africa and South Asia, continue to bear the highest burden. Somalia had the highest 2021 rates (ASPR: 47.86; ASYR: 9.40/100,000). SDI correlated negatively with ASPR (R = −0.62, *p* < 0.001) and ASYR (R = −0.62, *p* < 0.001). The slope index showed reductions in decline rates for prevalence (−19.256 [95%CI: −26.992, −11.520] to −12.531 [−16.107, − 8.955]) and YLDs (−3.662 [−5.047, − 2.276] to −2.451 [−3.144, − 1.757]), though overlapping confidence intervals indicated non-significance. Projections suggest stable ASPR/ASYR by 2040 but rising absolute cases (489,983 prevalent cases; 85,491 YLDs).

**Conclusion:**

While public health interventions have reduced the global burden of DID caused by iodine deficiency, persistent inequalities in vulnerable regions demand urgent policy action: scaling up universal salt iodization programs in high-burden areas, integrating maternal nutrition education into primary healthcare systems, and prioritizing resource allocation to regions with stagnating SDI indices.

## 1 Introduction

Developmental intellectual disability (DID) is a mental disorder characterized by below-average intelligence or cognitive ability during childhood (1). Children with developmental disabilities, particularly those in low- and middle-income countries, often face stigma and negative societal attitudes and beliefs from birth (2). These challenges heighten their vulnerability to neglect, exploitation, and violence, as well as increase the risk of premature death, including infanticide (3, 4).

The etiology of DID is believed to involve a complex interplay of factors, including genetic predispositions (5); parental health and behaviors, such as smoking and alcohol consumption during pregnancy (6); complications during childbirth; maternal or early-life infections; and exposure to high levels of environmental toxins, such as lead (7). Beyond these factors, many leading causes of disability are linked to the complications of infections and nutritional deficiencies (8), including iodine deficiency (9). Iodine deficiency, a significant global health issue, disrupts the synthesis of thyroid hormones, leading to numerous diseases related to metabolism and growth (10). Liang et al. (11) reported the global burden of iodine deficiency. There were 8.08 million incident cases of iodine deficiency in 2021. However, although severe iodine deficiency has been eliminated in virtually all countries, populations across numerous nations remain at risk of mild-to-moderate iodine insufficiency (12–14). Iodine deficiency exhibits a particularly high prevalence among children and adolescents. According to the most recent evidence, approximately 12.1 million children were influenced by iodine deficiency in 2019 (15). These conditions pose a serious threat to physical health and development. Thyroid hormones influence nearly all tissues in the body and play an essential role in regulating and sustaining growth, development, and metabolism at various life stages (16). In early childhood, thyroid hormone deficiency can result in irreversible intellectual developmental disabilities due to its critical role in neurodevelopment (17).

Previous studies have documented the burden of iodine deficiency among children and the associated disability attributable to DID in this population (8, 18). Yang et al. (19) analyzed the global burden of DID caused by iodine deficiency across all age groups from 1990 to 2019, identifying a disproportionately high burden in regions with low socio-demographic index (SDI). However, their study did not provide specific insights into the burden of DID among children under the age of 15. To address this gap, the present study analyzes the burden, trends, and health inequalities associated with childhood developmental intellectual disability caused by iodine deficiency from 1990 to 2021, based on the Global Burden of Disease (GBD) Study 2021. The findings aim to inform public health policymakers, guiding resource allocation and global efforts to promote child health.

## 2 Methods

### 2.1 Overview

The study design was an observational ecological time-trend study with forecasting components. Utilizing secondary data from the GBD 2021, which provides a comprehensive evaluation of health loss associated with 369 diseases, injuries, and impairments, as well as 88 risk factors, across 204 countries and territories, employing the latest epidemiological data and refined standardized methods.

### 2.2 Data extraction

Estimation of DID burden caused by Iodine deficiency was downloaded from the GBD result tool^1^ and stratified into three age groups: 0–4 years, 5–9 years, and 10–14 years for analysis. SDI is the geometric mean of the total fertility rate for individuals under age 25, the average age of 15 and above, and those with an education level of 15 and above, and per capita lagging distribution income. As a complex, a position with SDI 0 will have a theoretical minimum developmental level related to health, while a position with SDI 1 would have the highest level.

### 2.3 Burden description

This study recalculated the age-standardized rate (ASR) for the population aged 0–15 years. The calculation of the age-standardized rate was based on the standard population distribution of the World Health Organization (20), using the direct standardization method. The specific formula is as follows:


$$\mathrm{ASR}=(\frac{\sum_{i=1}^{n}w_{i}\cdot r_{i}}{\sum_{i=1}^{n}w_{i}})\times 100,000$$

Where *w_i_* is the weight of the *i*-th age group in the standard population, and *r_i_* is the age-specific rate.

The count, age-standardized prevalence rate, and age-standardized years lived with disability (YLD) rate (ASPR, ASYR) of DID caused by Iodine deficiency were reported at the global, regional, and national levels. Additionally, the description examines the demographic influence by comparing the genders (female, male, and both genders), age groups (categorized in 5-year intervals), and socioeconomic levels (high SDI, middle-high SDI, middle SDI, middle-low SDI, and low SDI). Descriptions of burden were with 95% uncertainty intervals (95% UI).

### 2.4 Trends analysis

Trend analysis mainly adopts the connection point regression method, which is a statistical method used to analyze trend changes in time series data by identifying “connection points,” dividing the time series into multiple stages, and calculating the average annual percent change (AAPC) and annual percent change (APC) of each segment and its 95% confidence interval (CI).

### 2.5 Health inequality assessment

The slope index of inequality (SII) was used to evaluate cross-country health inequality. The SII is an absolute measure of health inequality provided by the WHO Health Equity Assessment Toolkit Plus.13 In this study, the slope index of inequality represents the difference in estimated ASR between the country or territory with the lowest and highest SDI, accounting for population size through a regression model. The slope index is calculated by ranking a weighted sample of the population from the lowest to highest SDI. Greater absolute values of the slope index indicate higher levels of inequality.

### 2.6 BAPC prediction

Future trends in burden (2022–2050) were projected using a Bayesian age-period-cohort (BAPC) model. This approach accounts for age-specific effects, temporal trends (period), and birth cohort influences. Non-informative priors were applied to age, period, and cohort parameters. The posterior distributions were estimated via Markov Chain Monte Carlo simulations.

The R Software (version 4.2.2) was used to perform all analysis and visualization.

## 3 Results

### 3.1 Burden of DID due to Iodine deficiency in 2021

**Figure 1:**
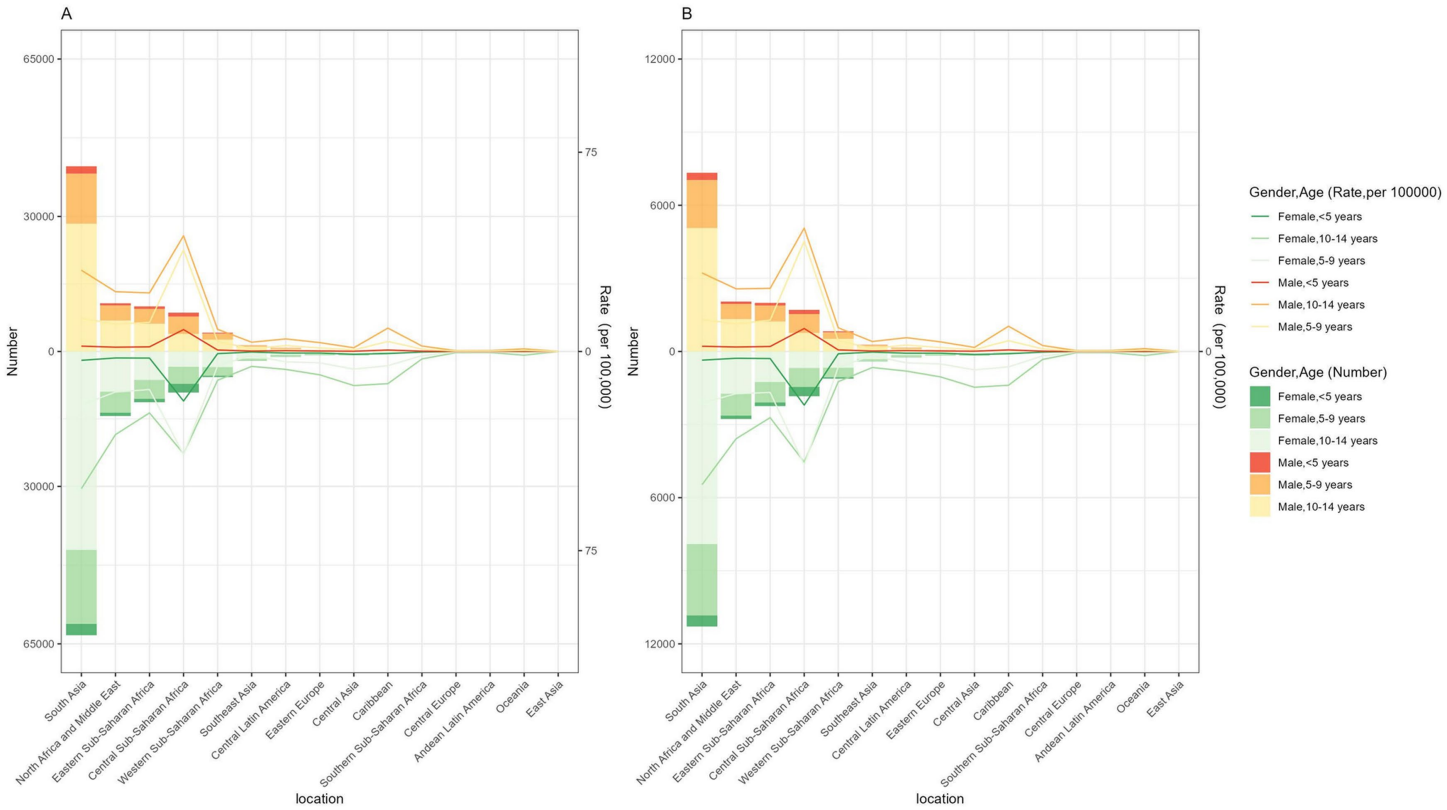
Number and rate of developmental intellectual disorder caused by Iodine deficiency among regions in 2021. **(A)** Prevalence; **(B)** YLD. YLD: Years lived with disability.

In five SDI regions, the highest-burden occurred in regions with low and low-middle SDI, followed by high and middle SDI. In contrast, the lowest was found in high SDI (Table 1). ASPR and ASYR of DID due to Iodine deficiency in 2021 were 8.96 cases (95% CI: 5.31–12.69) per 100,000 and 1.67 YLDs (95% CI: 0.82–2.79) per 100,000 (Table 1). Further, among all age groups, people aged 10–14 had the highest prevalence and YLDs. In contrast, children under five always had the lowest figures of these estimates (Figure 1).

**Table 1:** Burden of developmental intellectual disorder caused by Iodine deficiency in 2021 and trends from 1990 to 2021.

Location	Prevalence			YLDs		
	Number	ASPR	AAPC	Number	ASYR	AAPC
**Global**	186,825 (110874–264,394)	8.96 (5.31–12.69)	−4.95 (−5.52 to −4.36)	34,878 (17134–58,206)	1.67 (0.82–2.79)	−4.81 (−5.36 to −4.26)
SDI quintile						
High SDI	202 (6–339)	0.11 (0–0.18)	−1.57 (−1.74 to −1.39)	41 (1–74)	0.02 (0–0.04)	−1.57 (−1.74 to −1.39)
High-middle SDI	4,850 (1227–7,648)	1.97 (0.5–3.11)	−3.06 (−3.31 to −2.8)	968 (191–1885)	0.39 (0.08–0.77)	−2.96 (−3.25 to −2.67)
Middle SDI	10,013 (4745–15,051)	1.66 (0.79–2.5)	−8.47 (−9.77 to −7.14)	1979 (790–3,502)	0.33 (0.13–0.58)	−8.21 (−9.34 to −7.06)
Low-middle SDI	77,533 (46936–109,328)	12.87 (7.79–18.16)	−6.31 (−6.95 to −5.66)	14,163 (7171–23,718)	2.35 (1.19–3.94)	−6.21 (−6.85 to −5.57)
Low SDI	94,159 (54861–135,108)	20.76 (12.1–29.77)	−3.71 (−3.93 to −3.49)	17,714 (8516–29,562)	3.9 (1.88–6.51)	−3.55 (−3.79 to −3.32)
GBD super regions						
Central Europe, Eastern Europe, and Central Asia	2,136 (251–3,521)	2.52 (0.3–4.16)	−0.64 (−0.83 to −0.44)	423 (33–828)	0.5 (0.04–0.98)	−0.65 (−0.81 to −0.49)
Latin America and Caribbean	2,624 (157–4,637)	1.77 (0.11–3.12)	−0.97 (−3.44 to 1.56)	528 (27–1,082)	0.36 (0.02–0.73)	−0.95 (−3.41 to 1.57)
North Africa and Middle East	25,054 (12224–40,124)	13.37 (6.53–21.42)	−1.99 (−2.2 to −1.78)	4,819 (2077–8,376)	2.57 (1.11–4.47)	−1.96 (−2.17 to −1.75)
South Asia	104,248 (66501–145,199)	19.13 (12.18–26.69)	−6.44 (−7.19 to −5.68)	18,620 (10053–30,553)	3.42 (1.84–5.61)	−6.41 (−7.17 to −5.65)
Southeast Asia, East Asia, and Oceania	3,521 (1827–5,565)	0.75 (0.39–1.18)	−6.48 (−13.9 to 1.58)	700 (295–1,294)	0.15 (0.06–0.28)	−6.96 (−14.89 to 1.7)
Sub-Saharan Africa	49,241 (11414–78,025)	10.53 (2.44–16.7)	−1.65 (−1.75 to −1.55)	9,789 (2063–18,148)	2.09 (0.44–3.88)	−1.54 (−1.65 to −1.43)
GBD regions						
Central Asia	960 (251–1,483)	3.55 (0.92–5.49)	−2.29 (−2.59 to −1.98)	187 (27–389)	0.69 (0.1–1.44)	−2.3 (−2.53 to −2.06)
Central Europe	34 (0–61)	0.18 (0–0.32)	/*	7 (0–15)	0.04 (0–0.08)	/
Eastern Europe	1,141 (0–2016)	2.94 (0–5.21)	0.53 (0.19 to 0.87)	229 (0–479)	0.59 (0–1.24)	0.53 (0.22 to 0.84)
Andean Latin America	33 (12–58)	0.18 (0.06–0.31)	−5.6 (−5.84 to −5.36)	7 (2–13)	0.04 (0.01–0.07)	−5.6 (−5.83 to −5.36)
Caribbean	595 (121–1,010)	5.03 (1.03–8.54)	−0.91 (−1.05 to −0.77)	118 (18–253)	0.99 (0.15–2.14)	−0.96 (−1.2 to −0.71)
Central Latin America	1997 (8–3,659)	2.97 (0.01–5.45)	/	404 (1–838)	0.6 (0–1.25)	/
North Africa and Middle East	25,054 (12224–40,124)	13.37 (6.53–21.42)	−1.99 (−2.2 to −1.78)	4,819 (2077–8,376)	2.57 (1.11–4.47)	−1.96 (−2.17 to −1.75)
South Asia	104,248 (66501–145,199)	19.13 (12.18–26.69)	−6.44 (−7.19 to −5.68)	18,620 (10053–30,553)	3.42 (1.84–5.61)	−6.41 (−7.17 to −5.65)
East Asia	25 (9–47)	0.01 (0–0.02)	/	5 (2–10)	0 (0–0)	/
Oceania	27 (10–51)	0.56 (0.2–1.06)	−1.99 (−2.12 to −1.85)	5 (2–10)	0.11 (0.04–0.22)	−1.99 (−2.12 to −1.85)
Southeast Asia	3,469 (1803–5,476)	1.91 (0.99–3.02)	/	690 (289–1,275)	0.38 (0.16–0.7)	/
Central Sub-Saharan Africa	17,757 (3148–32,447)	30.52 (5.41–55.85)	0.33 (0.1 to 0.55)	3,544 (523–7,364)	6.09 (0.9–12.66)	0.35 (0.07 to 0.64)
Eastern Sub-Saharan Africa	21,304 (6015–33,184)	12.06 (3.4–18.78)	−2.39 (−2.57 to −2.21)	4,237 (1120–7,768)	2.4 (0.63–4.4)	−2.21 (−2.44 to −1.99)
Southern Sub-Saharan Africa	305 (0–569)	1.23 (0–2.3)	−3.57 (−3.95 to −3.18)	61 (0–134)	0.25 (0–0.54)	−3.6 (−4.11 to −3.09)
Western Sub-Saharan Africa	9,875 (1862–16,143)	4.78 (0.9–7.81)	−2.31 (−2.55 to −2.07)	1947 (322–3,564)	0.94 (0.16–1.72)	−2.35 (−2.64 to −2.07)
Countries/territories						
Afghanistan	4,378 (1895–8,037)	32.83 (14.26–60.15)	−0.04 (−0.34 to 0.27)	803 (245–1705)	6.02 (1.85–12.78)	−0.05 (−0.33 to 0.24)
Albania	12 (0–20)	2.5 (0–4.31)	/	2 (0–5)	0.5 (0–0.97)	/
Algeria	1,123 (528–1725)	8.63 (4.04–13.25)	−1.05 (−1.13 to −0.96)	224 (71–445)	1.72 (0.54–3.43)	−1.04 (−1.21 to −0.86)
Angola	2,128 (0–4,664)	14.23 (0–31.29)	−1.36 (−1.48 to −1.24)	430 (0–1,162)	2.87 (0–7.78)	−1.43 (−1.7 to −1.16)
Antigua and Barbuda	0 (0–1)	1.57 (0–3.15)	−2.38 (−2.49 to −2.27)	0 (0–0)	0.31 (0–0.69)	−2.38 (−2.49 to −2.27)
Bahamas	1 (0–3)	1.34 (0–2.87)	−1.15 (−1.22 to −1.08)	0 (0–1)	0.27 (0–0.63)	−1.15 (−1.22 to −1.08)
Bangladesh	5,060 (109–8,951)	10.45 (0.22–18.45)	−4.35 (−4.43 to −4.27)	1,001 (0–2,317)	2.07 (0–4.77)	−4.35 (−4.51 to −4.2)
Barbados	2 (0–4)	3.44 (0–6.67)	−0.69 (−0.88 to −0.5)	0 (0–1)	0.69 (0–1.45)	−0.69 (−0.88 to −0.5)
Belarus	28 (0–53)	1.68 (0–3.13)	−0.74 (−0.86 to −0.62)	6 (0–12)	0.34 (0–0.7)	−0.74 (−0.86 to −0.62)
Belize	2 (0–3)	1.2 (0–2.08)	−1.68 (−1.74 to −1.61)	0 (0–1)	0.24 (0–0.46)	−1.67 (−1.73 to −1.61)
Benin	419 (65–674)	7.38 (1.14–11.86)	−0.78 (−0.85 to −0.71)	82 (5–179)	1.45 (0.09–3.17)	−0.79 (−0.94 to −0.64)
Bermuda	0 (0–0)	0.67 (0–1.97)	−2.77 (−2.87 to −2.67)	0 (0–0)	0.14 (0–0.43)	−2.76 (−2.86 to −2.66)
Bolivia (Plurinational State of)	33 (12–58)	0.92 (0.33–1.64)	−0.89 (−0.92 to −0.86)	7 (2–13)	0.19 (0.05–0.37)	−0.89 (−0.92 to −0.86)
Bosnia and Herzegovina	22 (0–41)	4.2 (0–7.71)	/	4 (0–11)	0.83 (0–1.99)	/
Burkina Faso	736 (183–1,195)	7.7 (1.91–12.49)	−1.55 (−1.78 to −1.33)	147 (24–302)	1.53 (0.25–3.17)	−1.54 (−1.86 to −1.22)
Burundi	853 (351–1,347)	15.1 (6.19–23.84)	−1.83 (−2.3 to −1.37)	169 (37–375)	2.99 (0.66–6.64)	−1.83 (−2.31 to −1.36)
Cambodia	128 (65–209)	2.45 (1.25–4.01)	/	26 (12–47)	0.49 (0.22–0.9)	/
Cameroon	778 (76–1,280)	5.92 (0.57–9.73)	−0.27 (−0.35 to −0.19)	153 (5–327)	1.16 (0.04–2.49)	−0.22 (−0.41 to −0.04)
Central African Republic	759 (204–1,236)	33.95 (9.08–55.29)	0.13 (−0.05 to 0.3)	150 (34–310)	6.71 (1.5–13.87)	0.14 (−0.05 to 0.33)
Chad	698 (156–1,132)	8.57 (1.91–13.88)	−2.71 (−3.35 to −2.07)	135 (24–288)	1.66 (0.29–3.56)	−2.68 (−3.27 to −2.09)
Colombia	494 (0–971)	4.42 (0–8.69)	/	100 (0–226)	0.89 (0–2.02)	/
Comoros	10 (2–16)	4.03 (0.84–6.45)	−0.69 (−0.78 to −0.6)	2 (0–4)	0.79 (0.06–1.7)	−0.75 (−1.11 to −0.4)
Coted’Ivoire	779 (24–1,325)	7.07 (0.22–12.03)	−1.03 (−1.12 to −0.93)	154 (1–332)	1.4 (0.01–3.02)	−1.08 (−1.27 to −0.88)
Cuba	62 (1–107)	3.27 (0.05–5.62)	−1.37 (−1.48 to −1.26)	13 (0–24)	0.66 (0.01–1.25)	−1.3 (−1.44 to −1.15)
Democratic People’s Republic of Korea	25 (9–47)	0.49 (0.17–0.91)	/	5 (2–10)	0.1 (0.03–0.19)	/
Democratic Republic of the Congo	14,790 (2941–26,347)	39.16 (7.79–69.85)	0.74 (0.5 to 0.98)	2,949 (454–6,243)	7.81 (1.2–16.53)	0.73 (0.45 to 1)
Djibouti	63 (0–130)	15.28 (0.02–31.66)	−0.25 (−0.33 to −0.16)	12 (0–30)	2.99 (0–7.2)	−0.31 (−0.44 to −0.17)
Dominica	0 (0–1)	2.79 (0–4.93)	−2.16 (−2.25 to −2.06)	0 (0–0)	0.56 (0–1.08)	−2.15 (−2.23 to −2.07)
Dominican Republic	86 (0–164)	2.93 (0–5.56)	−2.56 (−2.79 to −2.34)	17 (0–36)	0.59 (0–1.23)	−2.53 (−2.8 to −2.27)
El Salvador	105 (0–187)	5.62 (0–10)	/	21 (0–47)	1.14 (0–2.54)	/
Equatorial Guinea	35 (0–112)	5.77 (0–18.61)	−5.27 (−5.59 to −4.95)	7 (0–23)	1.17 (0–3.79)	−5.22 (−5.57 to −4.86)
Eritrea	121 (41–191)	4.91 (1.66–7.76)	−1.33 (−1.44 to −1.23)	24 (6–49)	0.98 (0.26–1.99)	−1.35 (−1.55 to −1.15)
Estonia	2 (0–5)	1 (0–2.21)	−1.27 (−1.42 to −1.12)	0 (0–1)	0.2 (0–0.49)	−1.27 (−1.43 to −1.12)
Ethiopia	9,787 (1740–16,852)	22.29 (3.96–38.37)	−3.14 (−3.3 to −2.98)	1958 (343–3,894)	4.46 (0.78–8.87)	−2.87 (−3.05 to −2.69)
Gabon	45 (0–120)	6.88 (0–18.41)	−0.34 (−0.4 to −0.28)	9 (0–27)	1.36 (0–4.18)	−0.34 (−0.45 to −0.22)
Gambia	139 (18–235)	14.18 (1.87–23.98)	−0.5 (−0.56 to −0.44)	27 (2–62)	2.8 (0.16–6.37)	−0.49 (−0.64 to −0.34)
Ghana	1,157 (1–2,142)	9.16 (0.01–16.96)	−1.9 (−1.98 to −1.83)	232 (0–583)	1.84 (0–4.61)	−1.83 (−2.03 to −1.63)
Grenada	1 (0–1)	2.31 (0–4.1)	−2.98 (−3.06 to −2.9)	0 (0–0)	0.46 (0–0.89)	−3 (−3.14 to −2.86)
Guatemala	290 (0–505)	5.6 (0–9.75)	/	59 (0–131)	1.13 (0–2.53)	/
Guinea	761 (112–1,279)	13.16 (1.92–22.12)	−1.88 (−2.13 to −1.63)	148 (8–335)	2.56 (0.14–5.79)	−1.9 (−2.08 to −1.71)
Guinea-Bissau	129 (22–212)	14.87 (2.5–24.47)	−1.12 (−1.16 to −1.08)	26 (2–57)	2.97 (0.18–6.57)	−1.14 (−1.26 to −1.03)
Guyana	10 (2–16)	4.5 (0.73–7.51)	−2.09 (−2.26 to −1.92)	2 (0–4)	0.87 (0.05–1.83)	−2.18 (−2.4 to −1.97)
Haiti	373 (107–639)	8.7 (2.5–14.92)	−0.87 (−1.03 to −0.71)	73 (14–164)	1.7 (0.32–3.83)	−0.89 (−1.18 to −0.61)
Honduras	258 (3–427)	7.63 (0.1–12.62)	/	52 (0–114)	1.54 (0–3.35)	/
India	76,754 (47636–105,704)	19.11 (11.86–26.34)	−7.15 (−8.03 to −6.27)	13,327 (6900–21,704)	3.32 (1.71–5.41)	−7.2 (−8.09 to −6.3)
Indonesia	95 (47–160)	0.13 (0.07–0.23)	/	19 (8–35)	0.03 (0.01–0.05)	/
Iran (Islamic Republic of)	877 (454–1,364)	4.14 (2.14–6.43)	−3.11 (−3.25 to −2.97)	174 (76–320)	0.82 (0.36–1.51)	−2.79 (−2.91 to −2.68)
Iraq	2,225 (1049–3,327)	15.75 (7.45–23.56)	−0.92 (−1.13 to −0.7)	434 (101–880)	3.07 (0.72–6.23)	−0.93 (−1.26 to −0.61)
Jamaica	21 (0–37)	3.28 (0–5.66)	−1.7 (−1.76 to −1.63)	4 (0–8)	0.66 (0–1.26)	−1.68 (−1.76 to −1.6)
Jordan	344 (178–531)	8.65 (4.48–13.35)	−1.01 (−1.11 to −0.9)	69 (24–141)	1.74 (0.61–3.5)	−0.95 (−1.13 to −0.77)
Kiribati	0 (0–1)	0.68 (0.25–1.29)	−1.88 (−2 to −1.76)	0 (0–0)	0.14 (0.05–0.27)	−1.88 (−2 to −1.76)
Latvia	3 (0–7)	0.88 (0–2.23)	−1.02 (−1.09 to −0.94)	1 (0–2)	0.18 (0–0.5)	−1.02 (−1.09 to −0.94)
Lesotho	83 (0–159)	12.64 (0–24.11)	−2.73 (−2.93 to −2.53)	17 (0–43)	2.54 (0–6.48)	−2.8 (−3.14 to −2.45)
Liberia	140 (65–222)	6.36 (2.98–10.1)	−0.47 (−0.78 to −0.17)	27 (7–58)	1.23 (0.3–2.64)	−0.5 (−0.85 to −0.15)
Libya	257 (88–389)	15.31 (5.35–23.2)	0.78 (0.64 to 0.93)	50 (11–103)	2.99 (0.67–6.14)	0.69 (0.51 to 0.87)
Lithuania	4 (0–9)	0.91 (0–2.17)	−1.02 (−1.13 to −0.91)	1 (0–2)	0.18 (0–0.48)	−1.02 (−1.13 to −0.92)
Madagascar	1,505 (350–2,402)	12.84 (2.98–20.49)	0.94 (0.74 to 1.13)	302 (42–638)	2.57 (0.36–5.44)	0.96 (0.8 to 1.12)
Malawi	1,194 (337–1924)	14.2 (4.05–22.92)	−0.68 (−0.89 to −0.47)	237 (24–497)	2.82 (0.28–5.92)	−0.65 (−0.94 to −0.36)
Malaysia	205 (59–346)	2.54 (0.73–4.29)	−1.54 (−1.64 to −1.45)	41 (9–77)	0.51 (0.11–0.95)	−1.54 (−1.65 to −1.43)
Mali	732 (164–1,178)	6.88 (1.53–11.05)	−2.04 (−2.1 to −1.99)	143 (17–299)	1.34 (0.16–2.82)	−2.04 (−2.09 to −1.98)
Marshall Islands	0 (0–0)	0.57 (0.2–1.07)	−1.28 (−1.33 to −1.23)	0 (0–0)	0.11 (0.04–0.23)	−1.31 (−1.53 to −1.09)
Mauritania	211 (6–395)	11.49 (0.34–21.54)	/	42 (0–106)	2.32 (0–5.79)	/
Mexico	423 (0–780)	1.24 (0–2.28)	/	86 (0–195)	0.25 (0–0.57)	/
Micronesia (Federated States of)	0 (0–0)	0.58 (0.22–1.11)	−2 (−2.06 to −1.95)	0 (0–0)	0.12 (0.04–0.24)	−2 (−2.05 to −1.95)
Mongolia	47 (2–75)	4.5 (0.19–7.25)	−2.83 (−2.95 to −2.71)	9 (0–21)	0.89 (0–2)	−2.75 (−2.95 to −2.55)
Morocco	2058 (959–3,075)	20.33 (9.5–30.41)	−2.22 (−2.29 to −2.16)	402 (125–805)	3.98 (1.23–7.95)	−2.28 (−2.36 to −2.19)
Mozambique	1,137 (408–1784)	8.24 (2.94–12.93)	−3.84 (−4.3 to −3.37)	227 (53–458)	1.64 (0.38–3.33)	−3.66 (−4.1 to −3.22)
Myanmar	313 (150–525)	1.93 (0.92–3.24)	/	63 (28–112)	0.39 (0.17–0.69)	/
Namibia	28 (0–63)	3.35 (0–7.41)	−1.8 (−1.98 to −1.62)	6 (0–14)	0.67 (0–1.67)	−1.81 (−1.97 to −1.66)
Nicaragua	149 (1–249)	7.25 (0.06–12.14)	/	30 (0–63)	1.48 (0–3.06)	/
Niger	1868 (630–2,976)	16.08 (5.33–25.56)	−2.12 (−2.52 to −1.71)	369 (77–784)	3.17 (0.67–6.72)	−2.07 (−2.47 to −1.66)
Oman	73 (21–115)	6.12 (1.74–9.61)	−1 (−1.06 to −0.93)	15 (3–28)	1.22 (0.26–2.36)	−0.98 (−1.11 to −0.85)
Pakistan	22,435 (12253–35,006)	26.22 (14.32–40.91)	−0.32 (−0.98 to 0.34)	4,292 (1902–7,616)	5.02 (2.22–8.9)	−0.32 (−1.02 to 0.38)
Papua New Guinea	21 (7–40)	0.57 (0.2–1.1)	−2.2 (−2.28 to −2.12)	4 (1–8)	0.12 (0.04–0.22)	−2.2 (−2.28 to −2.11)
Philippines	1930 (919–2,964)	5.41 (2.57–8.33)	/	382 (149–716)	1.07 (0.42–2.01)	/
Puerto Rico	5 (0–13)	0.99 (0–2.45)	−2.5 (−2.96 to −2.03)	1 (0–3)	0.2 (0–0.54)	−2.5 (−2.95 to −2.04)
Republic of Moldova	7 (0–13)	1.26 (0–2.2)	0.67 (0.48 to 0.86)	1 (0–3)	0.25 (0–0.48)	0.66 (0.47 to 0.85)
Russian Federation	605 (0–1,101)	2.15 (0–3.92)	0.97 (0.69 to 1.25)	122 (0–250)	0.43 (0–0.89)	0.96 (0.68 to 1.25)
Saint Kitts and Nevis	0 (0–0)	1.5 (0–2.99)	−3.06 (−3.1 to −3.01)	0 (0–0)	0.3 (0–0.67)	−3.06 (−3.11 to −3.01)
Saint Lucia	0 (0–1)	1.44 (0–2.51)	−1.59 (−1.67 to −1.52)	0 (0–0)	0.29 (0–0.55)	−1.59 (−1.67 to −1.52)
Saint Vincent and the Grenadines	1 (0–1)	2.65 (0.1–4.64)	−2.64 (−2.71 to −2.56)	0 (0–0)	0.53 (0.02–1.02)	−2.62 (−2.76 to −2.47)
Sao Tome and Principe	5 (0–9)	6.15 (0.1–10.47)	−0.96 (−1.06 to −0.87)	1 (0–2)	1.26 (0–2.67)	−0.92 (−1.18 to −0.65)
Saudi Arabia	187 (6–312)	2.37 (0.08–3.94)	−1.08 (−1.17 to −1)	38 (1–69)	0.48 (0.01–0.87)	−1.08 (−1.16 to −1)
Senegal	602 (34–1,043)	9.55 (0.55–16.54)	−1.32 (−1.5 to −1.15)	118 (0–267)	1.86 (0–4.24)	−1.41 (−1.54 to −1.28)
Sierra Leone	324 (89–506)	9.45 (2.6–14.75)	−0.59 (−0.77 to −0.41)	64 (9–134)	1.87 (0.26–3.91)	−0.61 (−0.92 to −0.31)
Solomon Islands	1 (1–3)	0.55 (0.2–1.05)	−1.16 (−1.24 to −1.08)	0 (0–1)	0.11 (0.04–0.22)	−1.15 (−1.22 to −1.08)
Somalia	4,559 (2019–7,437)	47.86 (21.06–77.51)	−0.86 (−1.11 to −0.61)	895 (281–1726)	9.4 (2.96–18.04)	−0.69 (−0.88 to −0.51)
South Africa	194 (0–363)	1.23 (0–2.3)	−3.53 (−3.83 to −3.23)	39 (0–87)	0.24 (0–0.55)	−3.58 (−3.95 to −3.2)
South Sudan	447 (66–725)	10.49 (1.56–17)	0.64 (0.57 to 0.7)	90 (10–206)	2.12 (0.24–4.84)	0.6 (0.47 to 0.74)
Sudan	3,989 (1894–7,771)	23.63 (11.21–46.07)	−3.22 (−3.39 to −3.06)	755 (237–1,630)	4.47 (1.4–9.65)	−2.95 (−3.11 to −2.8)
Suriname	4 (0–6)	2.38 (0–4.15)	−2.17 (−2.28 to −2.07)	1 (0–1)	0.48 (0–0.93)	−2.18 (−2.28 to −2.07)
Syrian Arab Republic	784 (394–1,229)	18.03 (9.11–28.32)	−0.84 (−1.02 to −0.65)	152 (47–304)	3.5 (1.05–7.04)	−0.95 (−1.18 to −0.72)
Tajikistan	276 (102–426)	8.07 (2.96–12.46)	−0.37 (−0.53 to −0.21)	54 (8–118)	1.58 (0.24–3.44)	−0.4 (−0.59 to −0.2)
Thailand	217 (108–356)	1.96 (0.97–3.24)	/	43 (19–79)	0.39 (0.17–0.72)	/
Timor-Leste	9 (5–16)	1.79 (0.87–3)	/	2 (1–3)	0.36 (0.16–0.65)	/
Togo	398 (87–654)	12.2 (2.66–20.03)	−1.66 (−1.79 to −1.53)	78 (9–169)	2.39 (0.27–5.18)	−1.74 (−1.94 to −1.54)
Tokelau	0 (0–0)	0.49 (0.16–0.94)	−2.29 (−2.34 to −2.24)	0 (0–0)	0.1 (0.03–0.2)	−2.28 (−2.33 to −2.24)
Tonga	0 (0–0)	0.52 (0.17–1)	−2.02 (−2.07 to −1.97)	0 (0–0)	0.11 (0.03–0.22)	−2.02 (−2.07 to −1.96)
Trinidad and Tobago	6 (0–15)	1.86 (0–4.95)	−3.56 (−3.82 to −3.3)	1 (0–3)	0.38 (0–1.13)	−3.55 (−3.84 to −3.27)
Tunisia	157 (82–242)	5.46 (2.86–8.43)	−1.62 (−1.65 to −1.59)	31 (11–59)	1.08 (0.38–2.06)	−1.6 (−1.73 to −1.47)
Turkey	2,807 (742–4,353)	14.08 (3.76–21.83)	−2.31 (−2.44 to −2.18)	559 (85–1,189)	2.8 (0.43–5.96)	−2.28 (−2.46 to −2.11)
Tuvalu	0 (0–0)	0.57 (0.2–1.09)	−2.64 (−2.69 to −2.58)	0 (0–0)	0.11 (0.04–0.23)	−2.62 (−2.68 to −2.56)
Ukraine	492 (0–867)	6.71 (0–11.81)	0.41 (0.15 to 0.67)	99 (0–213)	1.35 (0–2.89)	0.53 (0.29 to 0.77)
United Republic of Tanzania	997 (129–1,622)	4.18 (0.54–6.8)	−1.21 (−1.33 to −1.09)	197 (8–422)	0.83 (0.03–1.77)	−1.24 (−1.5 to −0.97)
United States Virgin Islands	0 (0–0)	0.89 (0–2.2)	−3.12 (−3.39 to −2.86)	0 (0–0)	0.18 (0–0.49)	−3.12 (−3.38 to −2.86)
Uzbekistan	637 (135–1,013)	6.66 (1.41–10.6)	−1.78 (−1.86 to −1.69)	123 (7–290)	1.29 (0.07–3.03)	−1.86 (−2.03 to −1.69)
Vanuatu	3 (1–5)	2.31 (1.01–4.18)	−1.29 (−1.31 to −1.26)	1 (0–1)	0.46 (0.19–0.93)	−1.29 (−1.31 to −1.26)
Venezuela (Bolivarian Republic of)	278 (0–593)	4.04 (0–8.62)	/	56 (0–135)	0.81 (0–1.97)	/
Viet Nam	567 (254–933)	2.24 (1.01–3.69)	/	114 (44–214)	0.45 (0.18–0.85)	/
Yemen	5,772 (2814–10,512)	41.24 (20.11–75.19)	0.77 (0.25 to 1.29)	1,108 (408–2,103)	7.92 (2.91–15.04)	0.29 (−0.58 to 1.18)
Zambia	613 (26–1,027)	7.49 (0.31–12.55)	−1.18 (−1.29 to −1.08)	120 (1–262)	1.47 (0.01–3.2)	−1.19 (−1.41 to −0.98)

*: “/” indicates that AAPC could not be calculated due to incomplete data between 1990 and 2021. Values in parentheses represent 95% uncertainty intervals. AAPC: Average annual percentage change; ASPR: Age-standardized prevalence rate (per 100,000 population); ASYR: Age-standardized years lived with disability rate (per 100,000 population); SDI: Socio-demographic Index; YLDs: Years lived with disability.

In five SDI regions, the highest-burden occurred in regions with low SDI and low-middle SDI, followed by high-middle SDI and middle SDI, while the lowest was found in high SDI (Table 1). At the regional level, in 2021, the highest ASPR and ASYR of DID due to Iodine deficiency were in Central Sub-Saharan Africa, followed by South Asia (Table 1; Figure 1). However, East Asia showed the lowest ASPR and ASYR in 2021. Regarding the sex, the prevalence of DID due to Iodine deficiency was more considerable among females than males, so as the YLDs (Figure 1).

**Figure 2:**
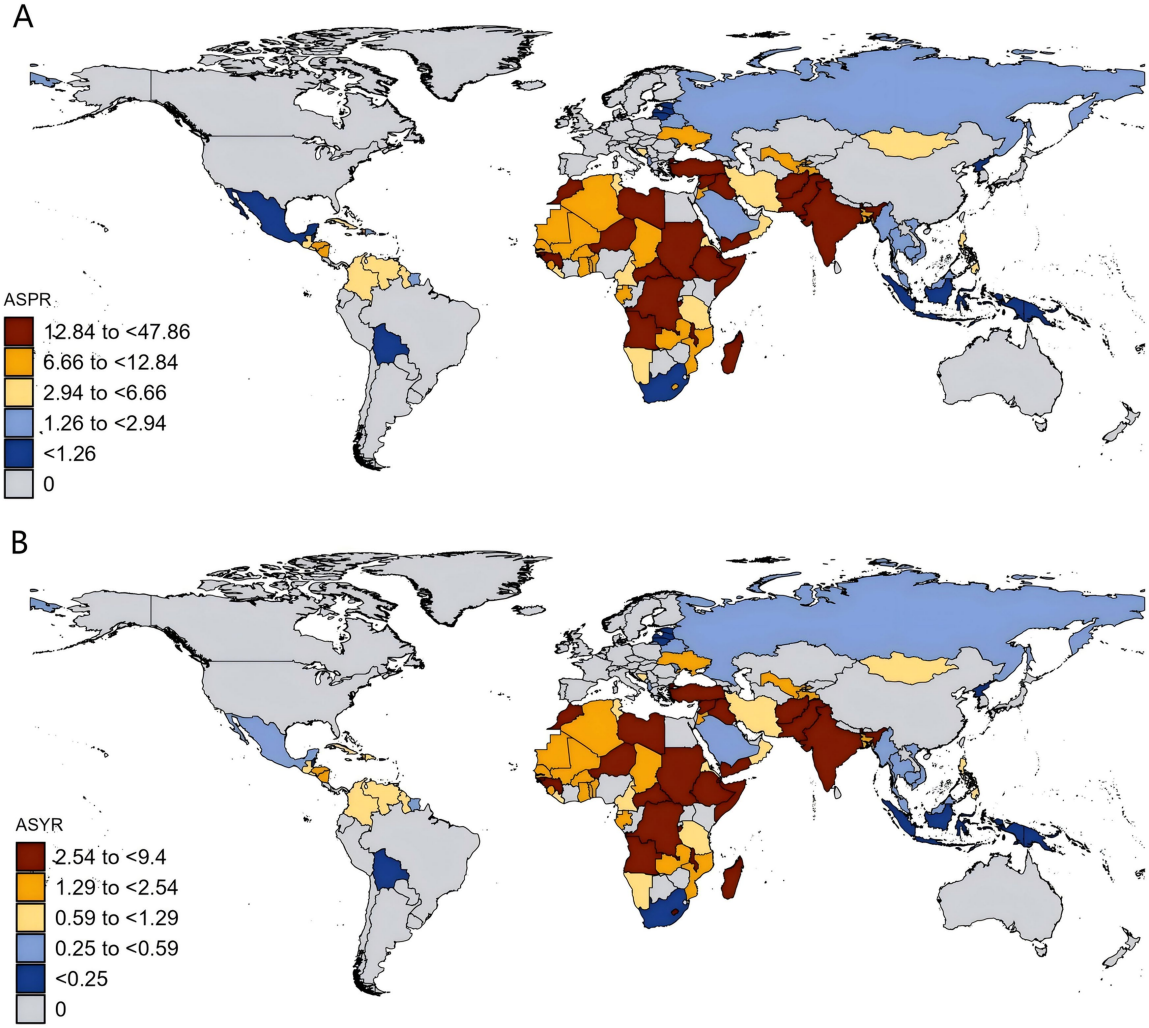
Age-standardized rates of developmental intellectual disorder caused by Iodine deficiency among countries/territories in 2021. **(A)** Prevalence; **(B)** YLD. YLD: Years lived with disability. ASPR: Age-standardized prevalence rate; ASYR: Age-standardized years lived with disability rate.

At the national level, the ASPR attributable to DID due to Iodine deficiency in 2021 varies among countries. The ASPR varied from 0.13/100,000 in Indonesia to 47.86/100,000 in Somalia (Figure 2; Table 1), of which five countries (Somalia, Yemen, the Democratic Republic of the Congo, Central African Republic, and Afghanistan) were over 30.00/100,000 and 16 countries were below 1.00/100,000. Similarly, Indonesia was also the lowest (0.03/100,000) about ASYR, while Somalia was the highest (9.40/100,000), followed by Yemen, Democratic Republic of the Congo, Central African Republic, Afghanistan, and Pakistan, where rates were estimated to exceed 5.00/100,000 (Figure 2; Table 1).

### 3.2 Trends of DID due to Iodine deficiency from 1990 to 2021

**Figure 3:**
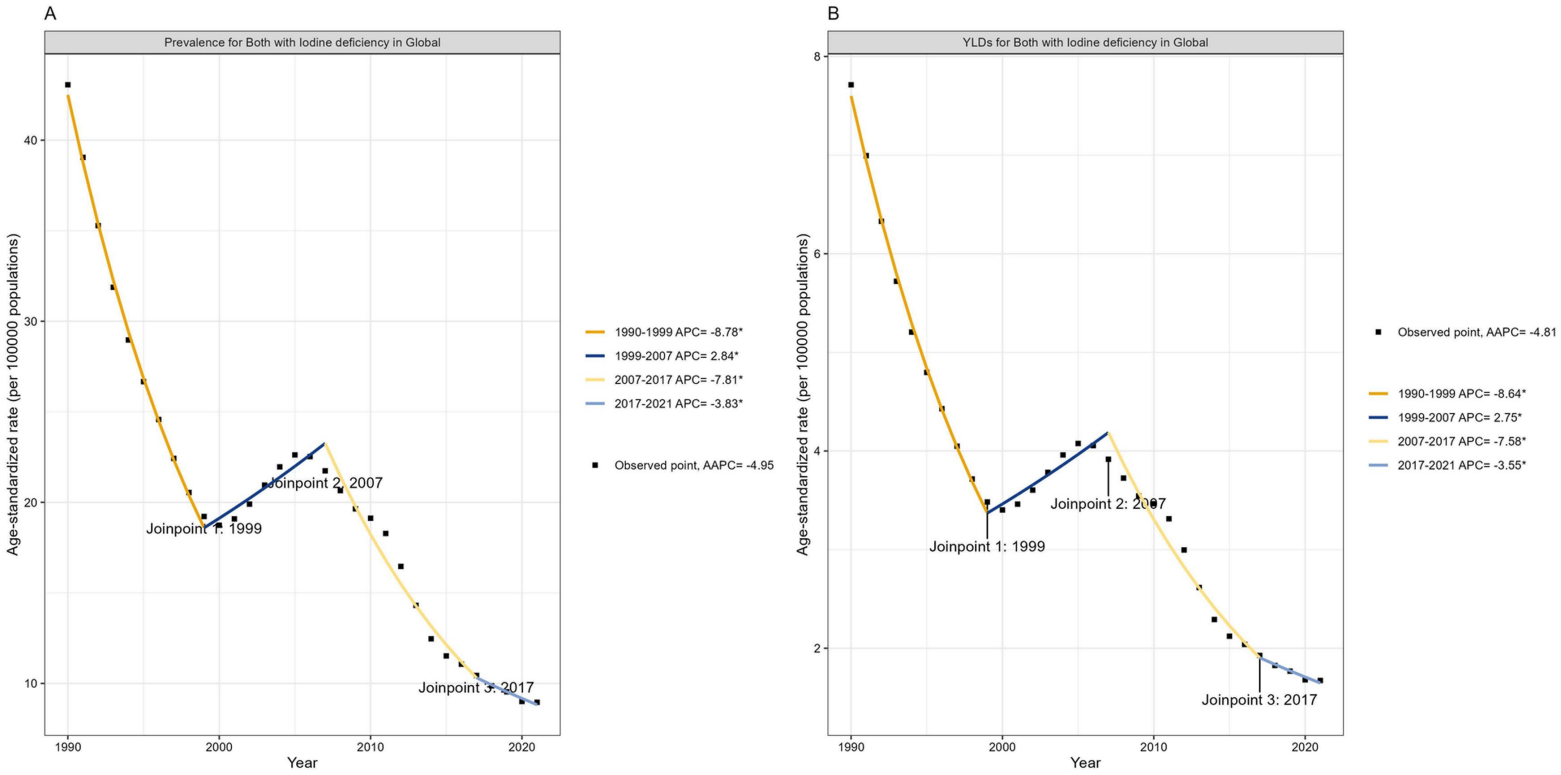
Trends in global developmental intellectual disorder caused by Iodine deficiency from 1990 to 2021. **(A)** Prevalence; **(B)** YLD. YLD: Years lived with disability.

**Figure 4:**
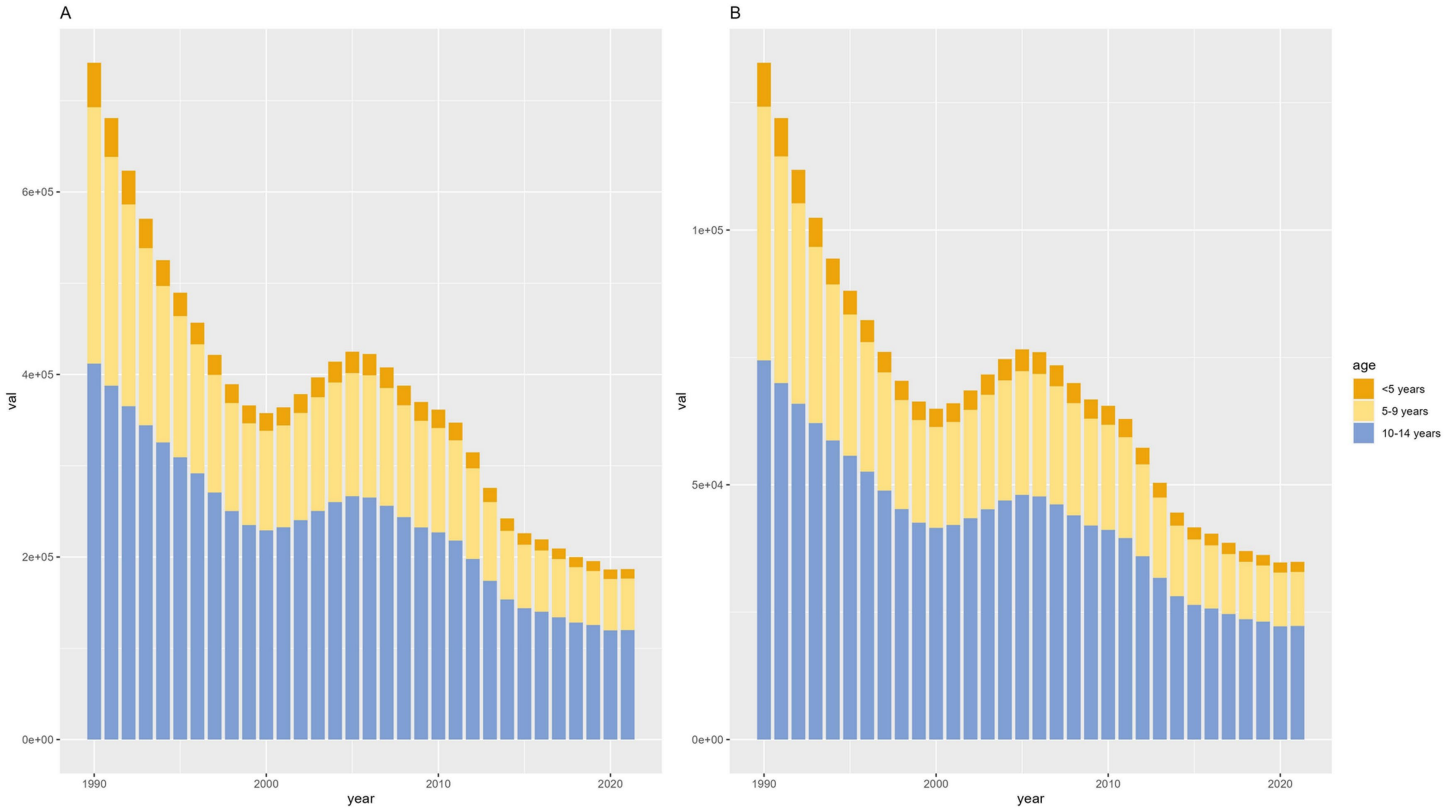
Counts of developmental intellectual disorder caused by Iodine deficiency between the ages of 1990 to 2021. **(A)** Prevalence; **(B)** YLD. YLD: Years lived with disability.

In the global analysis of 111 countries and regions, we evaluated the number of DID due to Iodine deficiency and ASR trends between 1990 and 2021. The annual cases number decreased from 741,573 in 1990 to 1868,825 in 2021, and the ASPR decreased from 43.06/100,000 in 1990 to 8.96/100,000 in 2021 (Table 1; Figures 3, 4), with an AAPC -4.95 (95% CI: −5.52 to −4.36). At the same time, the YLDs decreased from 132,831 in 1990 to 34,878 in 2021, and the ASYR generally showed a downward trend, from 7.71/100,000 in 1990 [95% UI 4.22–12.24] (Table 1) dropped to 1.67/100,000 in 2021 [95% UI 0.82–2.79] (Table 1), with an AAPC −4.81 (95% CI: −5.36 to −4.26). More specifically, as shown in Figure 3, ASPR and ASYR have significantly decreased from 1990 to 1999 (ASPR: APC = −8.78; ASYR: APC = −8.64); however ASPR and ASPR showed a slight upward trend from 1999 to 2007 (ASYR: APC = 2.84; ASYR = 2.75); from 2007 to 2017 (ASYR: APC = −7.81; ASPR: APC = −7.58) and from 2017 to 2021 (ASPR: APC = −3.83; ASYR: APC = −3.55), ASPR showed a significant downward trend.

According to different age groups, the annual number of diseases and the annual YLDs accounted for the most significant proportion in the 10-14-year-old group and the smallest in the <5-year-old group. Generally, from 1990 to 2021, the annual number of diseases and annual YLDs in different age groups showed a downward trend (Figure 4).

**Figure 5:**
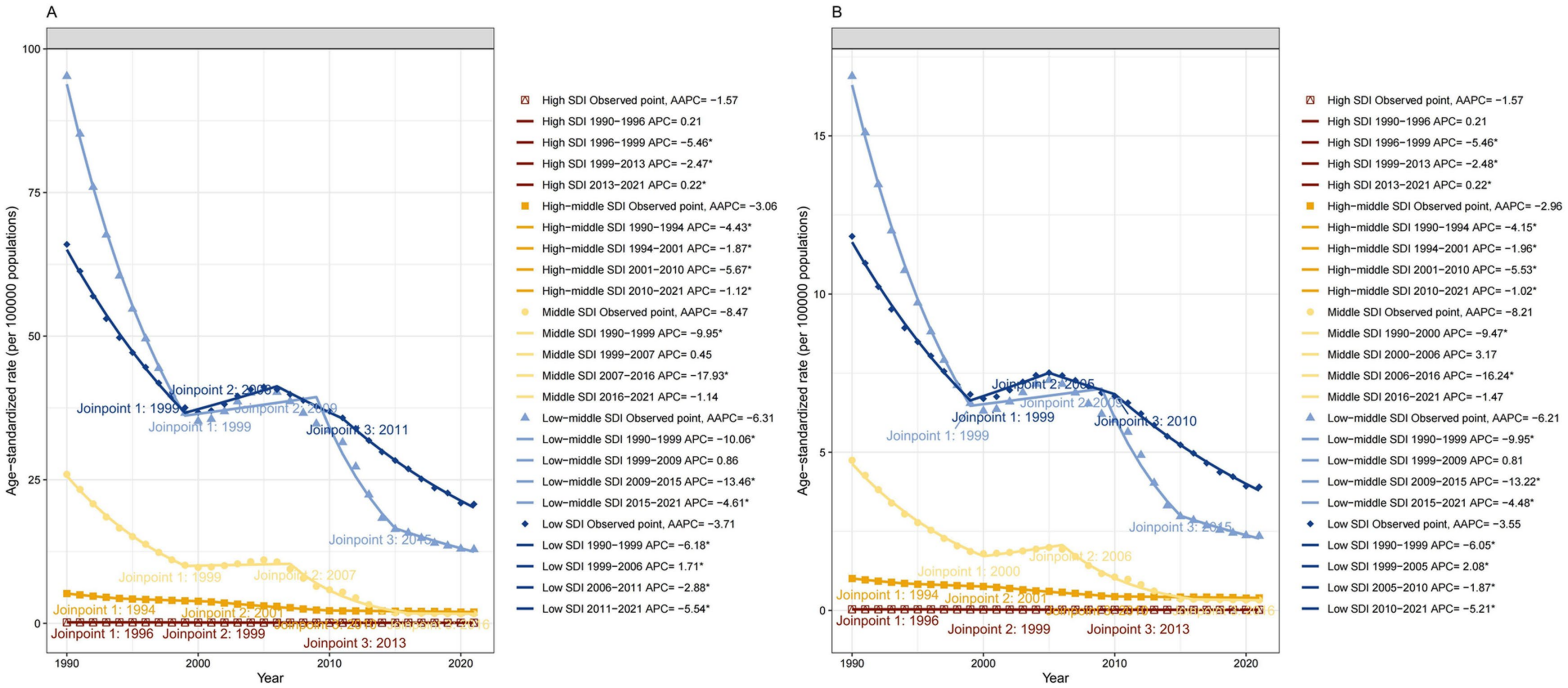
Trends in developmental intellectual disorder caused by Iodine deficiency among 5 SDI regions from 1990 to 2021. **(A)** Prevalence; **(B)** YLD. YLD: Years lived with disability.

At the regional level, the ASPR and ASYR of DID caused by Iodine deficiency decreased in most regions over the three decades. Significantly, South Asia showed the most significant decline from 1990 to 2021, with an AAPC of −6.44 (95% CI: −7.19 to −5.68) in ASPR and an AAPC of −6.41 (95% CI: −7.17 to −5.65) in ASYR. However, the DID burden in Eastern Europe and Central Sub-Saharan Africa was observed to be increasing at an abnormally high rate. Regarding the SDI quintile, between 1990 and 2021, the ASPR and ASYR of DID due to Iodine deficiency observed a most notable decline in Middle SDI regions, with an AAPC -8.47 (95% CI: −9.77 to −7.14). The burden was slightly decreased in high SDI regions but remained at the lowest level compared with other SDI regions (Figure 5).

At the national level, the most noticeable increase in the ASPR and ASDR of DID from 1990 to 2021 was observed in the Russian Federation, with an AAPC AAPCs of 0.97 (95% CI: 0.69 to 1.25) in ASPR and 0.96 (95% CI: 0.68 to 1.25) in ASDR The most apparent decrease of ASPR and ASDR in India, with an AAPC of −7.15 (95% CI: 0.69 to 1.25) in ASPR and −7.2 (95% CI: −8.09 to −6.3) in ASDR (Table 1).

### 3.3 Health inequality of DID due to iodine deficiency

**Figure 6:**
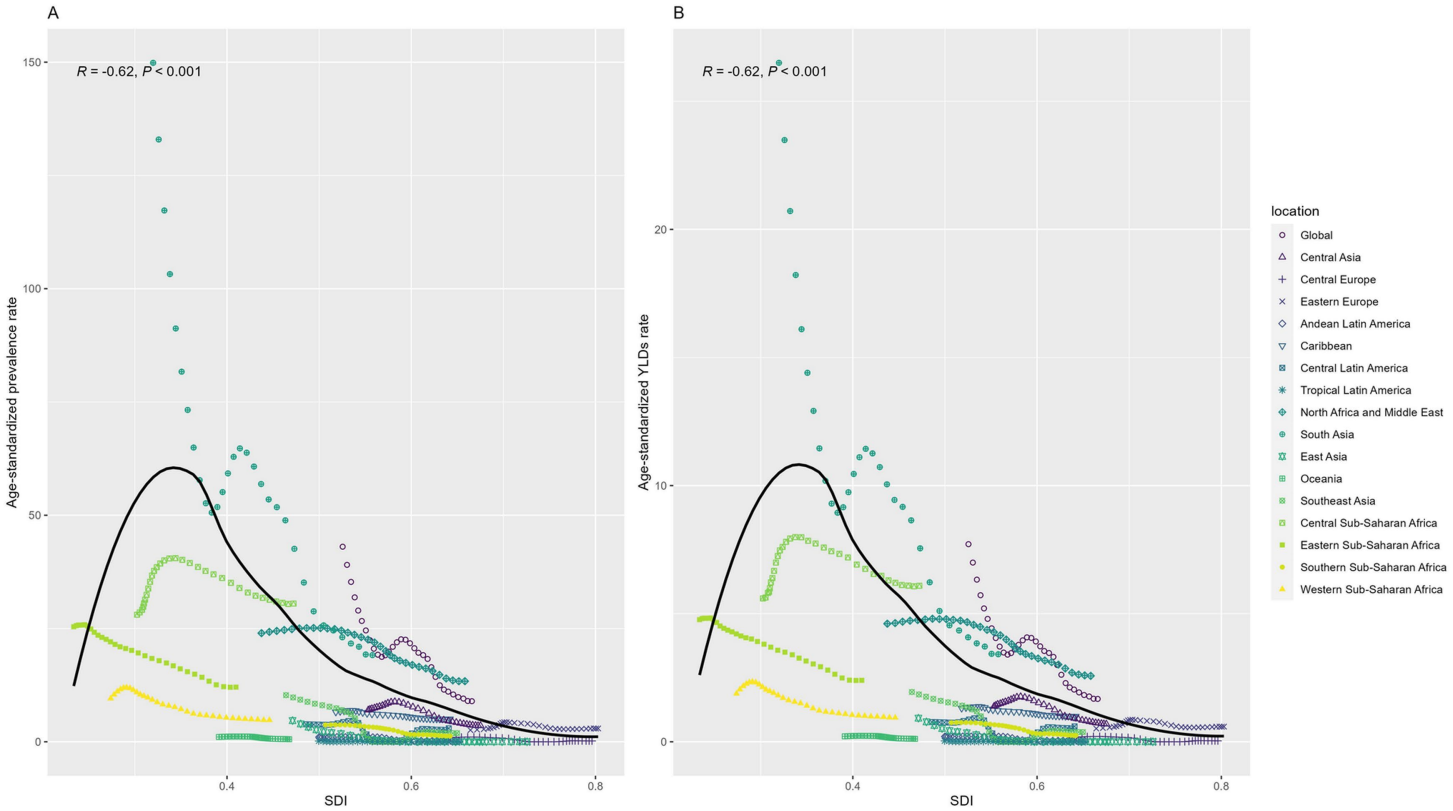
Relation between Age-standardized rates and SDI among regions from 1990 to 2021. **(A)** Prevalence; **(B)** YLD. YLD: Years lived with disability. SDI: socio-demographic index.

Figure 6 reflected a distinct inverted U-shaped relationship between the SDI and ASPR of DID. ASPR exhibited a rapid rise, peaking at an SDI of 0.3, followed by a gradual decline with further increases in SDI. Notably, a strong inverse correlation emerged between SDI and both ASPR (*R* = −0.62, *p* < 0.001) and ASYR (*R* = −0.62, *p* < 0.001).

**Figure 7:**
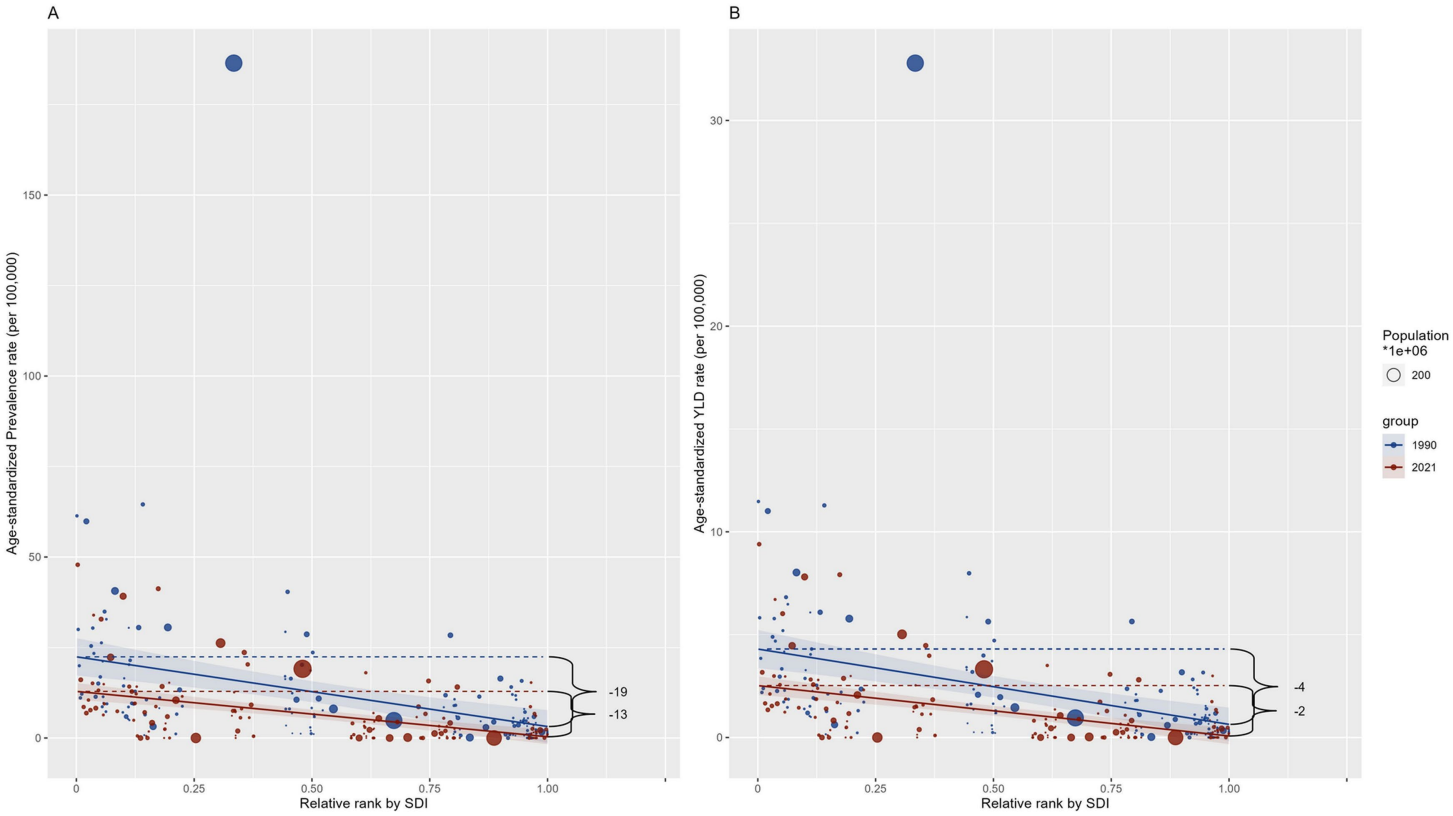
Slope index of ASRs among countries regarding the population and SDI in 1990 and 2021. **(A)** Prevalence; **(B)** YLD. YLD: Years lived with disability. SDI: socio-demographic index.

We further quantitatively evaluated the degree of health inequality of DID caused by Iodine deficiency regarding the SDI in 1990 and 2021 with the Slope index. In 1990 and 2021, the SII shows a decrease for both prevalence and YLDs from 1990 to 2021, with values shifting from −19.256 (95% CI -26.992 to −11.520) to −12.531 (95% CI -16.107 to −8.955) for prevalence and from −3.662 (95% CI −5.047 to −2.276) to −2.451 (95% CI -3.144 to −1.757) for YLDs, respectively (Figure 7). The decline suggests a reduction in the gap in the burden of DID between the highest and lowest SDI countries during this period. However, the difference is not statistically significant compared to the CI intervals.

### 3.4 The predicted burden of DID due to Iodine deficiency, 2022–2040

**Figure 8:**
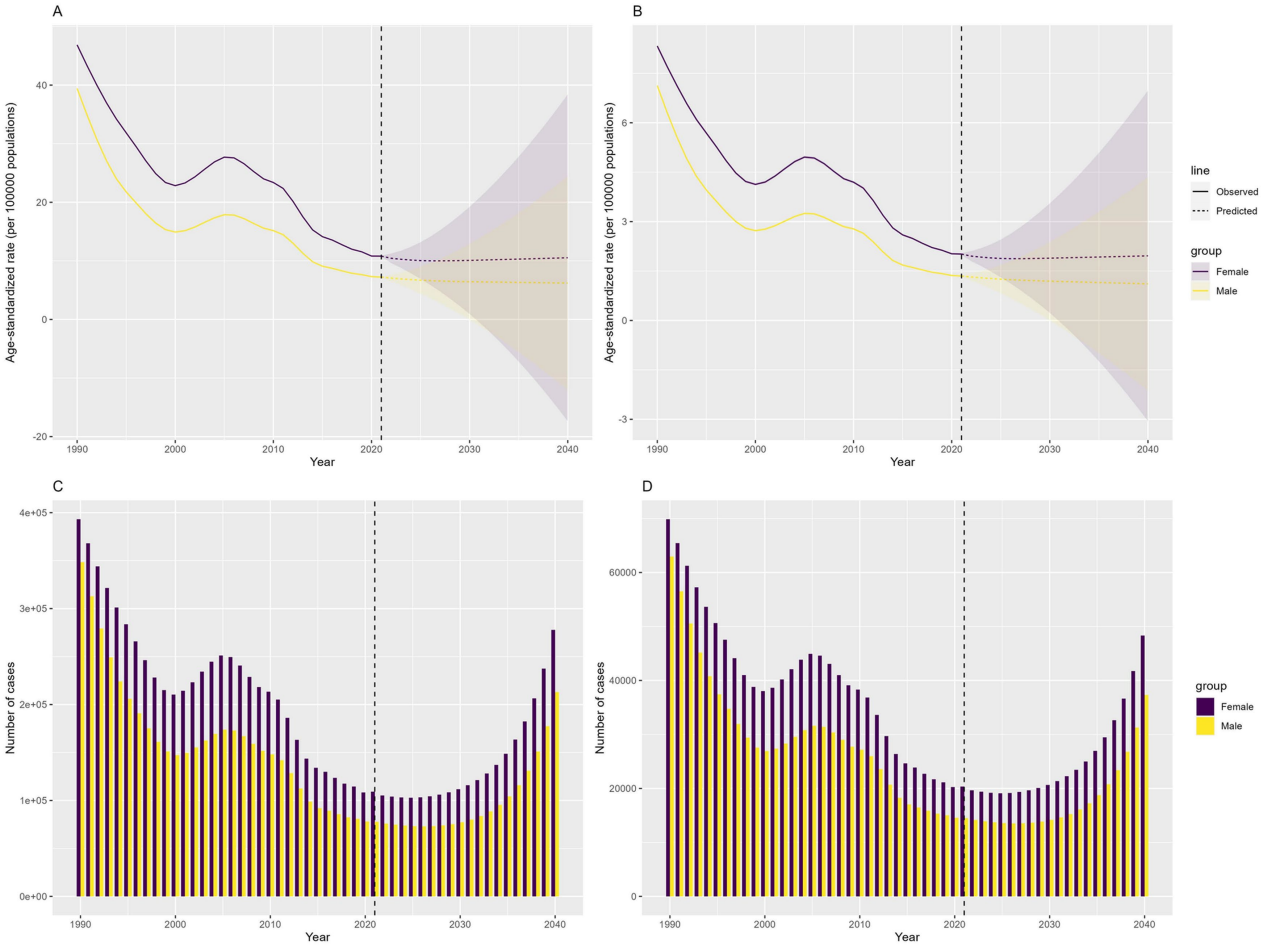
Predicted number and ASRs of the global burden of developmental intellectual disorder caused by Iodine deficiency from 2022 to 2040. **(A)** ASPR; **(B)** ASYR; **(C)** Prevalence number; **(D)** YLDs. YLD: Years lived with disability.

Prediction analysis using the BAPC model indicated a global increase in DID due to Iodine deficiency from 2022 to 2040. The prevalence of DID due to Iodine deficiency, which decreased from 741,570 in 1990 to 18,184 in 2021, is expected to increase to 489,983 in 2040. The YLDs of DID due to Iodine deficiency, which decreased from 132,827 in 1990 to 33,866 in 2021, is expected to rise to 85,491 in 2040. The ASPR and ASYR of DID due to Iodine deficiency are expected to remain stable (Figure 8).

## 4 Discussion

This new research reveals a striking 62% reduction in childhood DID caused by iodine deficiency from 1990 to 2021. Global prevalence rates and YLDs demonstrate steady decreases, with lower SDI regions achieving the most dramatic progress—sub-Saharan Africa saw a 63% drop in cases. Despite progress, alarming inequities persist: Low-SDI areas still carry a 10 times higher DID burden than high-income regions, highlighting urgent unmet needs.

Iodine deficiency disrupts the synthesis of thyroid hormones, which is critical for childhood neurodevelopment (21, 22). Moreover, insufficient iodine supply during pregnancy can lead to fetal brain damage (23, 24). Observational studies have shown that both low and high maternal iodine intake during pregnancy are associated with poorer neurodevelopmental outcomes in children (23, 25–27). A cohort study of 800 mother-infant pairs in Australia found that mean iodine intake during pregnancy below ~185 μg/day was associated with poorer cognitive and language scores in children at 18 months of age (28, 29). Therefore, the Declaration on the Survival, Protection, and Development of Children was formulated after the 1989 World Summit for Children held by the United Nations in New York, which states that every mother has the right to adequate iodine nutrition to ensure the normal brain development of newborns (30, 31).

However, in 2020, the diets of 21 countries worldwide were still deficient in iodine (32) particularly in developing countries. According to the WHO, 42.6% of the African population had low iodine intake (33). The negative correlation between SDI and the burden of iodine deficiency-related DID demonstrates a transparent socioeconomic gradient. The decline in the SII over the study period indicates progress in reducing global health disparities. Iodine exists in nature as iodide, which is present in seawater and evaporates into the atmosphere and returns to the soil. However, in inland areas, this cycle is incomplete, and the iodide in the soil has already been depleted (34). In central and eastern sub-Saharan Africa, insufficient access to iodized salt and limited dietary diversity have contributed to the increase in iodine deficiency. This is particularly true for rural populations, who rely on non-iodized salt, and where infrastructure to support sustained salt iodization remains weak (10). Previous studies have also observed the heavy burden of iodine deficiency in Africa (11, 35–37).

Liang et al. (11) employed extreme gradient boosting to predict the future trend of iodine deficiency and reported that the prevalence of iodine deficiency peaks at around 20–45 years. Similarly, our study, through BAPC prediction, also observed that the global DID caused by iodine deficiency will reach its peak in 2040. Wang and Feng (38) pointed out that malnutrition caused severe mental disability among children in underdeveloped regions. Our analysis aligns with established evidence confirming the outsized impact of iodine deficiency on childhood cognition across developing economies (7, 39).

Iodine supplementation programs have been initiated globally, including direct iodine supplementation and iodine fortification of foods. For example, as one of the countries severely affected by iodine deficiency disorders, China implemented the Universal Salt Iodization (USI) program in 1995, successfully eliminating iodine deficiency as a public health problem (40, 41). USI has reduced the incidence of iodine deficiency in most regions and has also contributed to reducing the burden of DID caused by iodine deficiency (12). Meanwhile, over the past three decades, the WHO, the UN, and other international organizations have prioritized efforts to eliminate hunger and malnutrition. The Food and Agriculture Organization of the United Nations (FAO) and the United Nations Children’s Fund have set global goals to reduce hunger and malnutrition (42). The Sustainable Development Goals (SDG) 2.2, which aim to end all forms of malnutrition and hunger, have also promoted the improvement of iodine deficiency in low-income regions (43).

However, the improvements remain modest and require further action. These stark contrasts highlight the critical role of socioeconomic development and health infrastructure in mitigating iodine deficiency-related health outcomes. Our study also reveals the implementation effect of USI in lower SDI regions, with the ASPR and ASYR showing a significant decline in low-SDI and low-middle SDI regions. Although the burden of DID caused by iodine deficiency has increased in Russia and Ukraine over the past 30 years, the study indicates that this may be attributed to the impact of the Chernobyl disaster (44, 45). Overall, it cannot be ignored that the negative slope index indicates that health inequalities still exist.

Thus, these results underscore the importance of targeted public health interventions to address iodine deficiency, particularly in regions with low SDI. Policymakers should take these findings into account to prioritize resource allocation and devise interventions aimed at promoting iodine supplementation and nutrition education, particularly in Central Sub-Saharan Africa and South Asia. This entails strengthening the implementation and monitoring of iodized salt distribution, especially in low SDI regions. Additionally, it is crucial to enhance public health campaigns by launching education programs targeting pregnant women and caregivers to raise awareness about the significance of iodine in early childhood development. Integrating iodine supplementation into maternal and child healthcare services in high-risk regions is also essential. Moreover, addressing broader socioeconomic factors through the promotion of development policies that improve education, healthcare access, and poverty reduction is necessary to foster an enabling environment for better health outcomes.

## 5 Strengths and Limitations

The present study offers significant strengths. First, to our knowledge, this study is the first comprehensive global analysis of childhood DID burden attributable to iodine deficiency. Second, our findings derive credibility from the use of standardized methodologies and open-access data from the GBD study, ensuring reproducibility while minimizing the ethical concerns associated with primary data collection. Additionally, the integration of projections to 2040, coupled with explicit quantification of disparities across SDI regions, delivers actionable evidence for prioritizing interventions in high-burden regions.

Despite these strengths, several limitations warrant consideration. First, as an observational ecological study, our design precludes causal inferences between iodine deficiency and DID outcomes, reflecting inherent constraints in secondary data analysis. Second, while GBD data provides global coverage, potential misestimation may arise from heterogeneous diagnostic criteria across regions, unmeasured confounders (e.g., genetic predispositions, environmental toxins, or sociocultural factors influencing iodine uptake), and aggregated reporting that precludes individual-level analysis. Finally, our 2040 projections rely on current epidemiological patterns; unexpected shifts in interventions such as salt iodization policies, socioeconomic disruptions, or emergent comorbidities could alter burden trajectories not captured by the Bayesian model.

## 6 Conclusion

The findings underscore the significant decline in the global burden of childhood DID attributable to iodine deficiency from 1990 to 2021, driven by improved public health interventions, such as USI and SDG 2.2. However, substantial disparities persist, with low SDI regions, particularly Central Sub-Saharan Africa and South Asia, continuing to bear a disproportionately high burden. Policymakers should prioritize expanding USI, enhancing nutrition education, and addressing broader socioeconomic determinants to mitigate health inequalities further.

To strengthen the evidence base, future research should investigate how local contextual factors influence intervention effectiveness across different regions, incorporate individual-level biomarker assessments, such as urinary iodine concentration, to obtain more precise estimates of deficiency impact, and prioritize prospective cohort studies that track individuals from prenatal stages through childhood.

## Data Availability

The original contributions presented in the study are included in the article/Supplementary material, further inquiries can be directed to the corresponding author.

## Footnotes

^1^ https://vizhub.healthdata.org/gbd-results/
